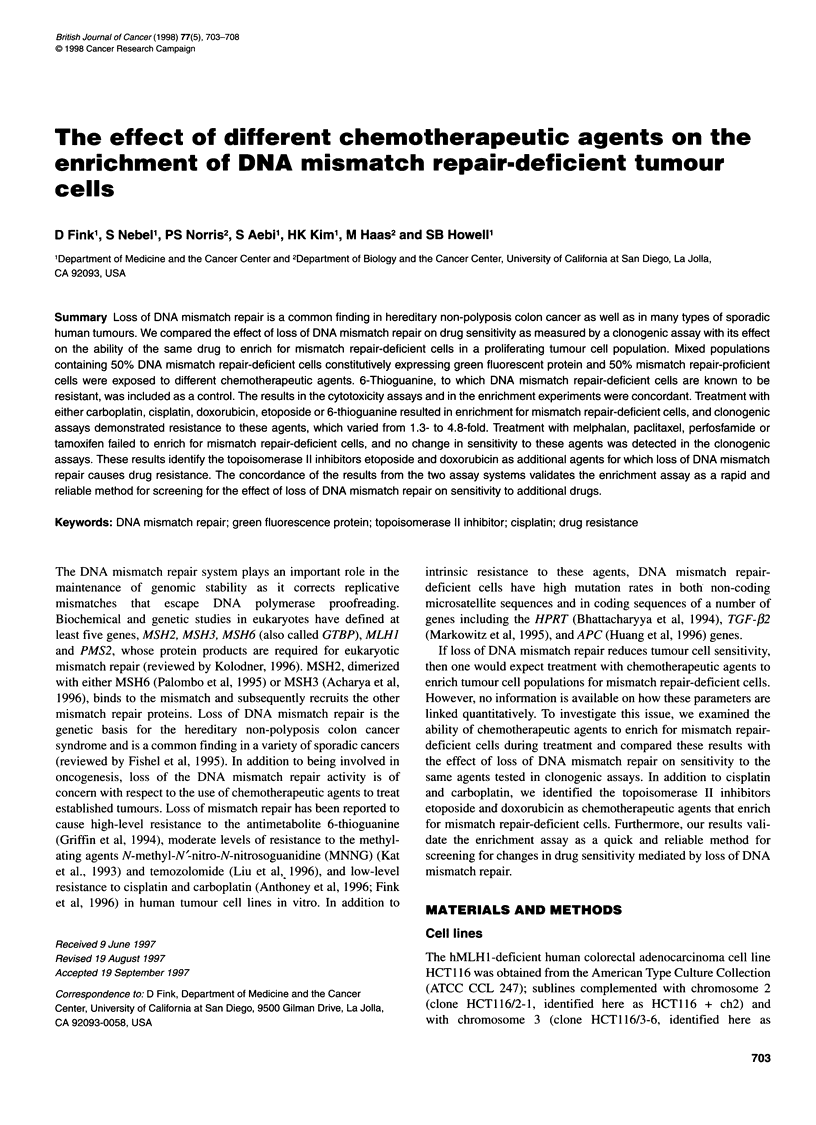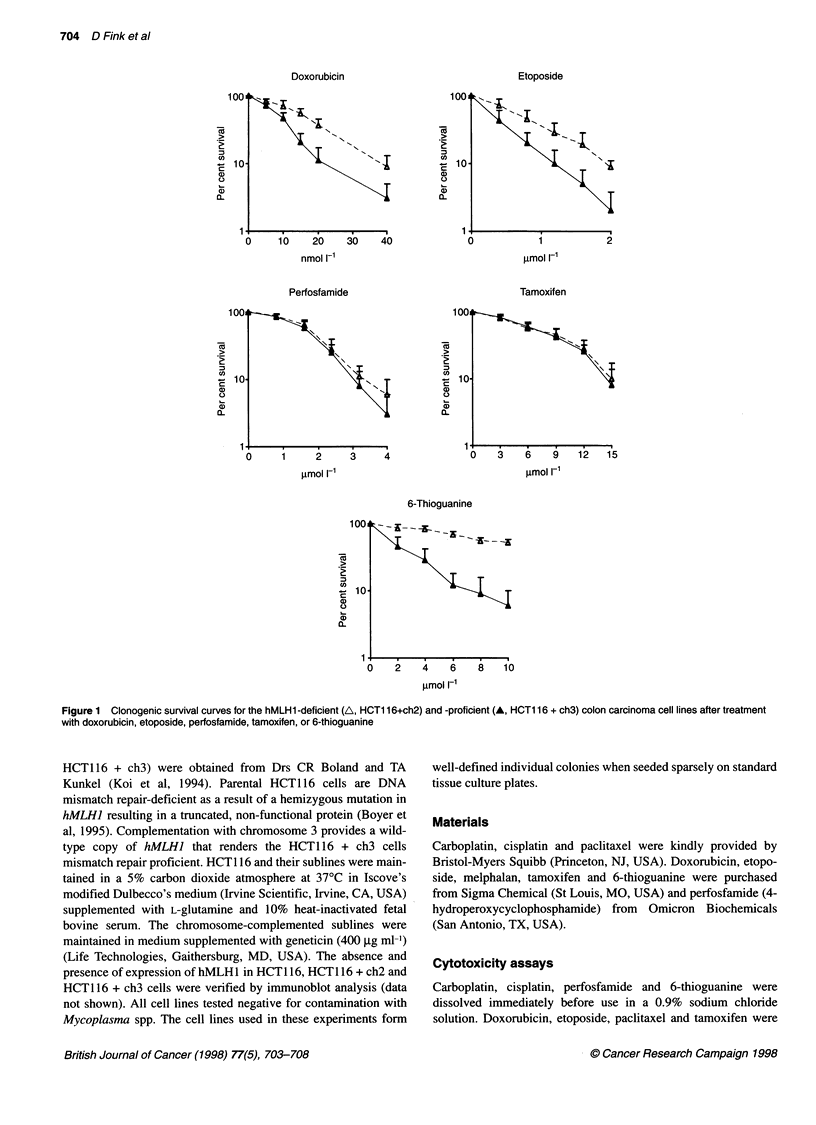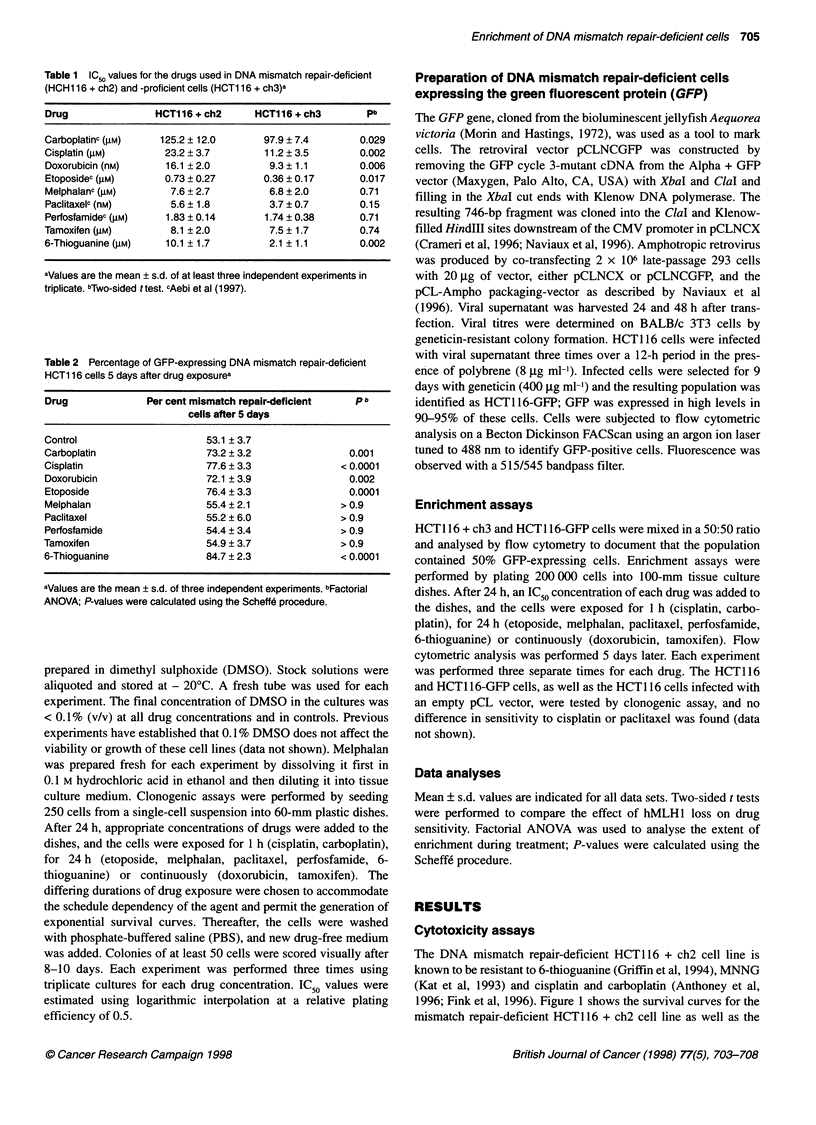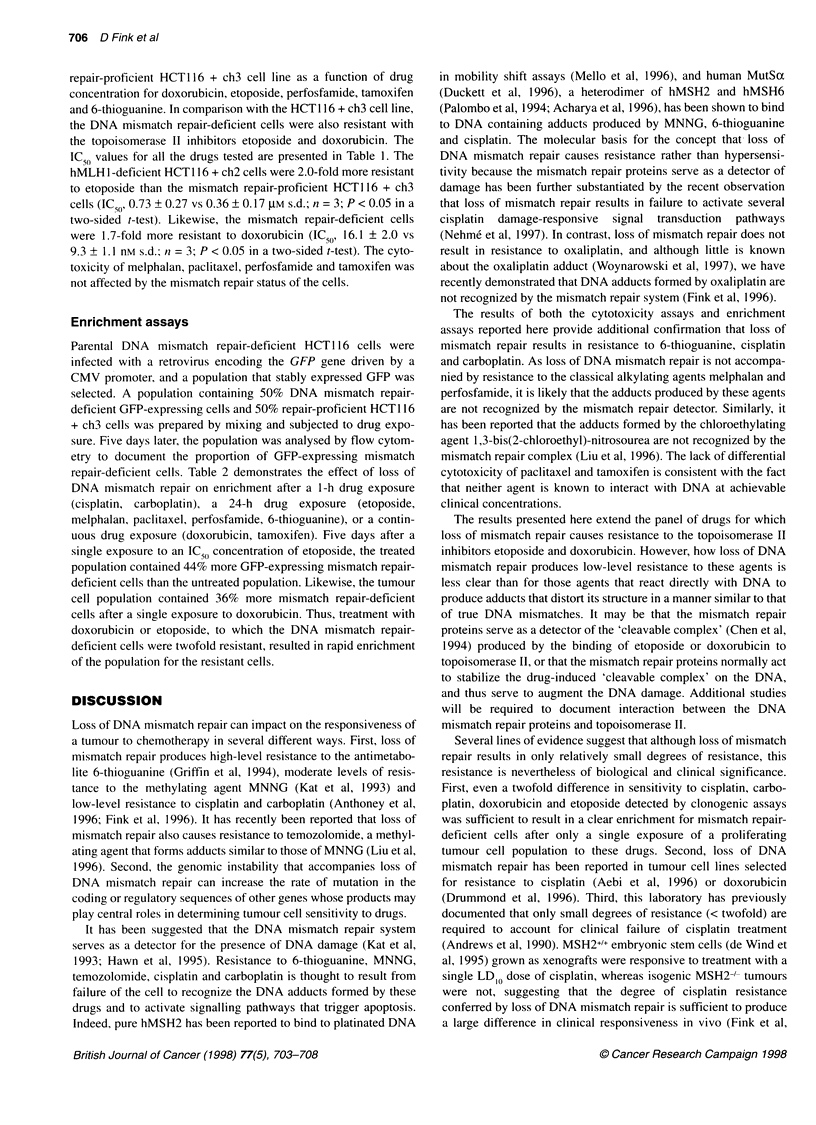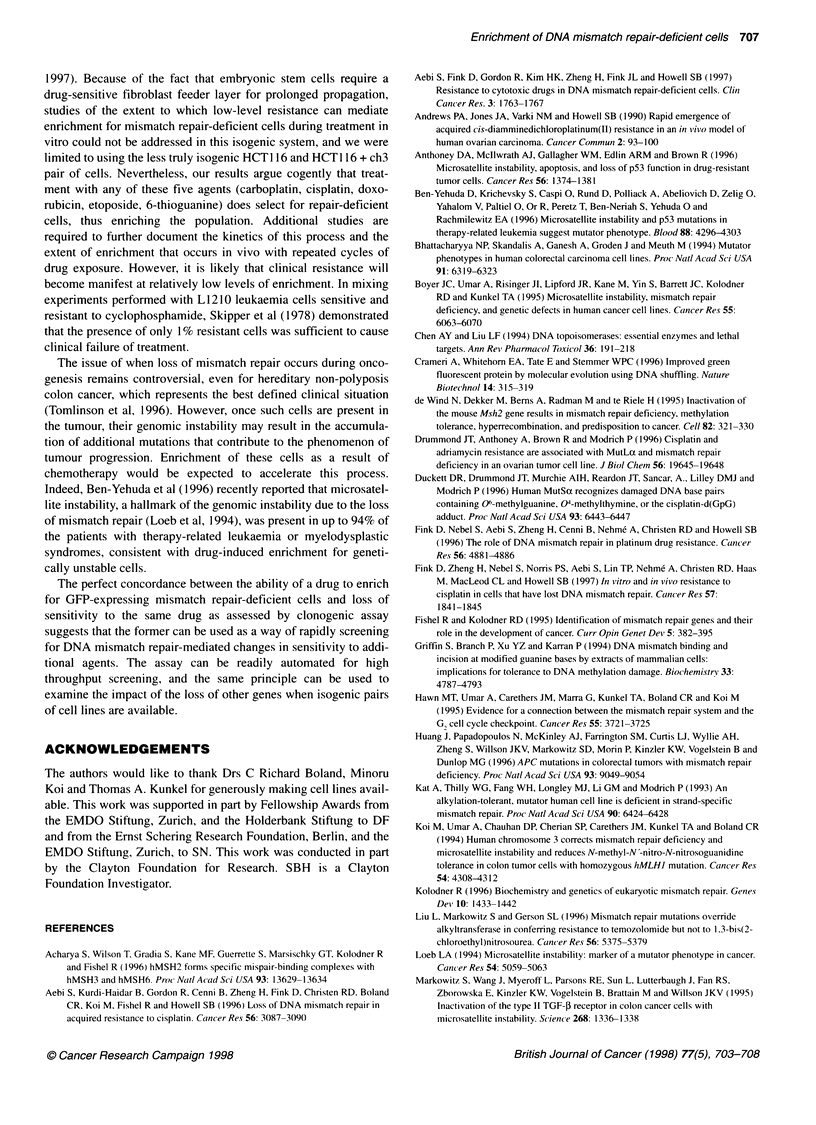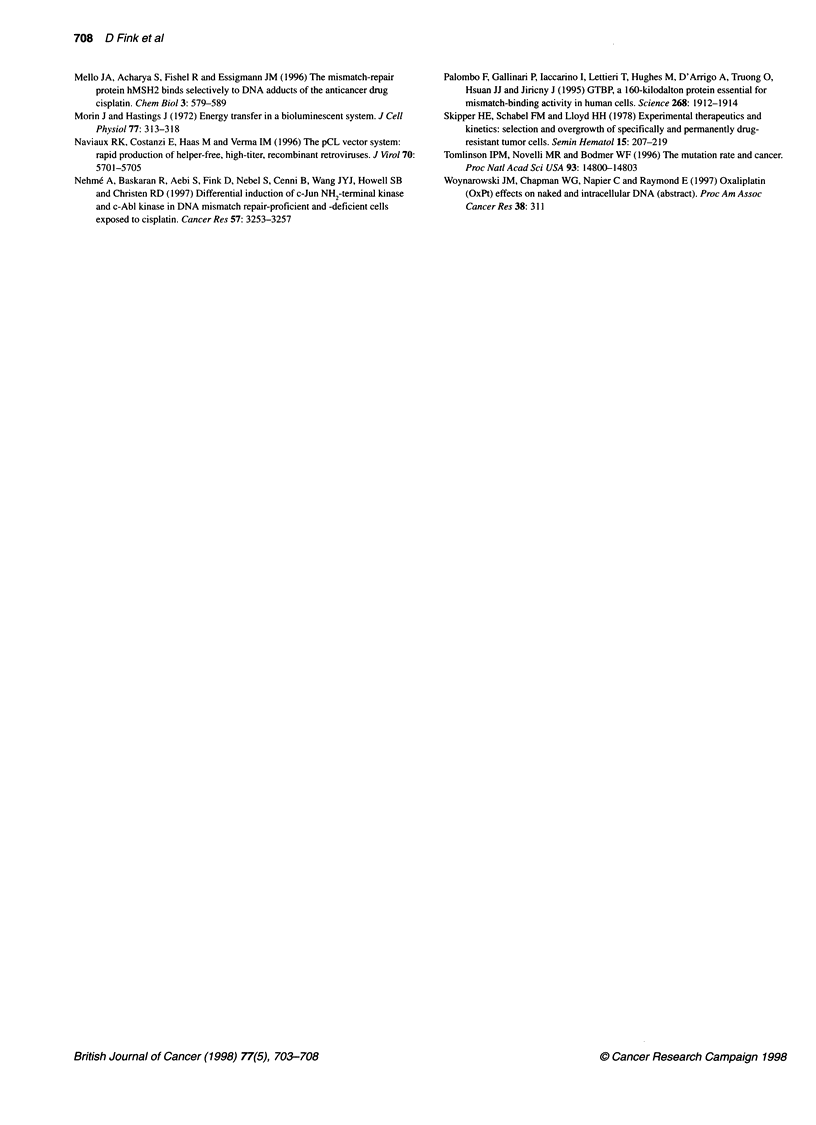# The effect of different chemotherapeutic agents on the enrichment of DNA mismatch repair-deficient tumour cells.

**DOI:** 10.1038/bjc.1998.116

**Published:** 1998-03

**Authors:** D. Fink, S. Nebel, P. S. Norris, S. Aebi, H. K. Kim, M. Haas, S. B. Howell

**Affiliations:** Department of Medicine and the Cancer Center, University of California at San Diego, La Jolla 92093-0058, USA.

## Abstract

Loss of DNA mismatch repair is a common finding in hereditary non-polyposis colon cancer as well as in many types of sporadic human tumours. We compared the effect of loss of DNA mismatch repair on drug sensitivity as measured by a clonogenic assay with its effect on the ability of the same drug to enrich for mismatch repair-deficient cells in a proliferating tumour cell population. Mixed populations containing 50% DNA mismatch repair-deficient cells constitutively expressing green fluorescent protein and 50% mismatch repair-proficient cells were exposed to different chemotherapeutic agents. 6-Thioguanine, to which DNA mismatch repair-deficient cells are known to be resistant, was included as a control. The results in the cytotoxicity assays and in the enrichment experiments were concordant. Treatment with either carboplatin, cisplatin, doxorubicin, etoposide or 6-thioguanine resulted in enrichment for mismatch repair-deficient cells, and clonogenic assays demonstrated resistance to these agents, which varied from 1.3- to 4.8-fold. Treatment with melphalan, paclitaxel, perfosfamide or tamoxifen failed to enrich for mismatch repair-deficient cells, and no change in sensitivity to these agents was detected in the clonogenic assays. These results identify the topoisomerase II inhibitors etoposide and doxorubicin as additional agents for which loss of DNA mismatch repair causes drug resistance. The concordance of the results from the two assay systems validates the enrichment assay as a rapid and reliable method for screening for the effect of loss of DNA mismatch repair on sensitivity to additional drugs.


British Journal of Cancer (1998) 77(5), 703–708
© 1998 Cancer Research Campaign

### The effect of different chemotherapeutic agents on the enrichment of DNA mismatch repair-deficient tumour cells

**D Fink[1], S Nebel[1], PS Norris[2], S Aebi[1], HK Kim[1], M Haas[2] and SB Howell[1]**

[1]Department of Medicine and the Cancer Center and [2]Department of Biology and the Cancer Center, University of California at San Diego, La Jolla, CA 92093, USA

**Summary** Loss of DNA mismatch repair is a common finding in hereditary non-polyposis colon cancer as well as in many types of sporadic human tumours. We compared the effect of loss of DNA mismatch repair on drug sensitivity as measured by a clonogenic assay with its effect on the ability of the same drug to enrich for mismatch repair-deficient cells in a proliferating tumour cell population. Mixed populations containing 50% DNA mismatch repair-deficient cells constitutively expressing green fluorescent protein and 50% mismatch repair-proficient cells were exposed to different chemotherapeutic agents. 6-Thioguanine, to which DNA mismatch repair-deficient cells are known to be resistant, was included as a control. The results in the cytotoxicity assays and in the enrichment experiments were concordant. Treatment with either carboplatin, cisplatin, doxorubicin, etoposide or 6-thioguanine resulted in enrichment for mismatch repair-deficient cells, and clonogenic assays demonstrated resistance to these agents, which varied from 1.3- to 4.8-fold. Treatment with melphalan, paclitaxel, perfosfamide or tamoxifen failed to enrich for mismatch repair-deficient cells, and no change in sensitivity to these agents was detected in the clonogenic assays. These results identify the topoisomerase II inhibitors etoposide and doxorubicin as additional agents for which loss of DNA mismatch repair causes drug resistance. The concordance of the results from the two assay systems validates the enrichment assay as a rapid and reliable method for screening for the effect of loss of DNA mismatch repair on sensitivity to additional drugs.

**Keywords:** DNA mismatch repair; green fluorescence protein; topoisomerase II inhibitor; cisplatin; drug resistance

The DNA mismatch repair system plays an important role in the maintenance of genomic stability as it corrects replicative mismatches that escape DNA polymerase proofreading. Biochemical and genetic studies in eukaryotes have defined at least five genes, *MSH2, MSH3, MSH6* (also called *GTBP*), *MLH1* and *PMS2*, whose protein products are required for eukaryotic mismatch repair (reviewed by Kolodner, 1996). MSH2, dimerized with either MSH6 (Palombo et al, 1995) or MSH3 (Acharya et al, 1996), binds to the mismatch and subsequently recruits the other mismatch repair proteins. Loss of DNA mismatch repair is the genetic basis for the hereditary non-polyposis colon cancer syndrome and is a common finding in a variety of sporadic cancers (reviewed by Fishel et al, 1995). In addition to being involved in oncogenesis, loss of the DNA mismatch repair activity is of concern with respect to the use of chemotherapeutic agents to treat established tumours. Loss of mismatch repair has been reported to cause high-level resistance to the antimetabolite 6-thioguanine (Griffin et al, 1994), moderate levels of resistance to the methylating agents *N*-methyl-*N*′-nitro-*N*-nitrosoguanidine (MNNG) (Kat et al., 1993) and temozolomide (Liu et al, 1996), and low-level resistance to cisplatin and carboplatin (Anthoney et al, 1996; Fink et al, 1996) in human tumour cell lines in vitro. In addition to intrinsic resistance to these agents, DNA mismatch repair-deficient cells have high mutation rates in both non-coding microsatellite sequences and in coding sequences of a number of genes including the *HPRT* (Bhattacharyya et al, 1994), *TGF-β2* (Markowitz et al, 1995), and *APC* (Huang et al, 1996) genes.

If loss of DNA mismatch repair reduces tumour cell sensitivity, then one would expect treatment with chemotherapeutic agents to enrich tumour cell populations for mismatch repair-deficient cells. However, no information is available on how these parameters are linked quantitatively. To investigate this issue, we examined the ability of chemotherapeutic agents to enrich for mismatch repair-deficient cells during treatment and compared these results with the effect of loss of DNA mismatch repair on sensitivity to the same agents tested in clonogenic assays. In addition to cisplatin and carboplatin, we identified the topoisomerase II inhibitors etoposide and doxorubicin as chemotherapeutic agents that enrich for mismatch repair-deficient cells. Furthermore, our results validate the enrichment assay as a quick and reliable method for screening for changes in drug sensitivity mediated by loss of DNA mismatch repair.

*Received 9 June 1997*
*Revised 19 August 1997*
*Accepted 19 September 1997*

*Correspondence to:* D Fink, Department of Medicine and the Cancer Center, University of California at San Diego, 9500 Gilman Drive, La Jolla, CA 92093-0058, USA

### MATERIALS AND METHODS

### Cell lines

The hMLH1-deficient human colorectal adenocarcinoma cell line HCT116 was obtained from the American Type Culture Collection (ATCC CCL 247); sublines complemented with chromosome 2 (clone HCT116/2-1, identified here as HCT116 + ch2) and with chromosome 3 (clone HCT116/3-6, identified here as

**Figure 1** Clonogenic survival curves for the hMLH1-deficient (△, HCT116+ch2) and -proficient (▲, HCT116 + ch3) colon carcinoma cell lines after treatment with doxorubicin, etoposide, perfosfamide, tamoxifen, or 6-thioguanine

HCT116 + ch3) were obtained from Drs CR Boland and TA Kunkel (Koi et al, 1994). Parental HCT116 cells are DNA mismatch repair-deficient as a result of a hemizygous mutation in *hMLH1* resulting in a truncated, non-functional protein (Boyer et al, 1995). Complementation with chromosome 3 provides a wild-type copy of *hMLH1* that renders the HCT116 + ch3 cells mismatch repair proficient. HCT116 and their sublines were maintained in a 5% carbon dioxide atmosphere at 37°C in Iscove's modified Dulbecco's medium (Irvine Scientific, Irvine, CA, USA) supplemented with L-glutamine and 10% heat-inactivated fetal bovine serum. The chromosome-complemented sublines were maintained in medium supplemented with geneticin (400 μg ml$^{-1}$) (Life Technologies, Gaithersburg, MD, USA). The absence and presence of expression of hMLH1 in HCT116, HCT116 + ch2 and HCT116 + ch3 cells were verified by immunoblot analysis (data not shown). All cell lines tested negative for contamination with *Mycoplasma* spp. The cell lines used in these experiments form well-defined individual colonies when seeded sparsely on standard tissue culture plates.

### Materials

Carboplatin, cisplatin and paclitaxel were kindly provided by Bristol-Myers Squibb (Princeton, NJ, USA). Doxorubicin, etoposide, melphalan, tamoxifen and 6-thioguanine were purchased from Sigma Chemical (St Louis, MO, USA) and perfosfamide (4-hydroperoxycyclophosphamide) from Omicron Biochemicals (San Antonio, TX, USA).

### Cytotoxicity assays

Carboplatin, cisplatin, perfosfamide and 6-thioguanine were dissolved immediately before use in a 0.9% sodium chloride solution. Doxorubicin, etoposide, paclitaxel and tamoxifen were

© Cancer Research Campaign 1998

**Table 1** $IC_{50}$ values for the drugs used in DNA mismatch repair-deficient (HCH116 + ch2) and -proficient cells (HCT116 + ch3)[a]

| Drug | HCT116 + ch2 | HCT116 + ch3 | P[b] |
|---|---|---|---|
| Carboplatin[c] (μM) | 125.2 ± 12.0 | 97.9 ± 7.4 | 0.029 |
| Cisplatin (μM) | 23.2 ± 3.7 | 11.2 ± 3.5 | 0.002 |
| Doxorubicin (nM) | 16.1 ± 2.0 | 9.3 ± 1.1 | 0.006 |
| Etoposide[c] (μM) | 0.73 ± 0.27 | 0.36 ± 0.17 | 0.017 |
| Melphalan[c] (μM) | 7.6 ± 2.7 | 6.8 ± 2.0 | 0.71 |
| Paclitaxel[c] (nM) | 5.6 ± 1.8 | 3.7 ± 0.7 | 0.15 |
| Perfosfamide[c] (μM) | 1.83 ± 0.14 | 1.74 ± 0.38 | 0.71 |
| Tamoxifen (μM) | 8.1 ± 2.0 | 7.5 ± 1.7 | 0.74 |
| 6-Thioguanine (μM) | 10.1 ± 1.7 | 2.1 ± 1.1 | 0.002 |

[a]Values are the mean ± s.d. of at least three independent experiments in triplicate. [b]Two-sided *t* test. [c]Aebi et al (1997).

**Table 2** Percentage of GFP-expressing DNA mismatch repair-deficient HCT116 cells 5 days after drug exposure[a]

| Drug | Per cent mismatch repair-deficient cells after 5 days | *P*[b] |
|---|---|---|
| Control | 53.1 ± 3.7 | |
| Carboplatin | 73.2 ± 3.2 | 0.001 |
| Cisplatin | 77.6 ± 3.3 | < 0.0001 |
| Doxorubicin | 72.1 ± 3.9 | 0.002 |
| Etoposide | 76.4 ± 3.3 | 0.0001 |
| Melphalan | 55.4 ± 2.1 | > 0.9 |
| Paclitaxel | 55.2 ± 6.0 | > 0.9 |
| Perfosfamide | 54.4 ± 3.4 | > 0.9 |
| Tamoxifen | 54.9 ± 3.7 | > 0.9 |
| 6-Thioguanine | 84.7 ± 2.3 | < 0.0001 |

[a]Values are the mean ± s.d. of three independent experiments. [b]Factorial ANOVA; *P*-values were calculated using the Scheffé procedure.

prepared in dimethyl sulphoxide (DMSO). Stock solutions were aliquoted and stored at – 20°C. A fresh tube was used for each experiment. The final concentration of DMSO in the cultures was < 0.1% (v/v) at all drug concentrations and in controls. Previous experiments have established that 0.1% DMSO does not affect the viability or growth of these cell lines (data not shown). Melphalan was prepared fresh for each experiment by dissolving it first in 0.1 M hydrochloric acid in ethanol and then diluting it into tissue culture medium. Clonogenic assays were performed by seeding 250 cells from a single-cell suspension into 60-mm plastic dishes. After 24 h, appropriate concentrations of drugs were added to the dishes, and the cells were exposed for 1 h (cisplatin, carboplatin), for 24 h (etoposide, melphalan, paclitaxel, perfosfamide, 6-thioguanine) or continuously (doxorubicin, tamoxifen). The differing durations of drug exposure were chosen to accommodate the schedule dependency of the agent and permit the generation of exponential survival curves. Thereafter, the cells were washed with phosphate-buffered saline (PBS), and new drug-free medium was added. Colonies of at least 50 cells were scored visually after 8–10 days. Each experiment was performed three times using triplicate cultures for each drug concentration. $IC_{50}$ values were estimated using logarithmic interpolation at a relative plating efficiency of 0.5.

### Preparation of DNA mismatch repair-deficient cells expressing the green fluorescent protein (*GFP*)

The *GFP* gene, cloned from the bioluminescent jellyfish *Aequorea victoria* (Morin and Hastings, 1972), was used as a tool to mark cells. The retroviral vector pCLNCGFP was constructed by removing the GFP cycle 3-mutant cDNA from the Alpha + GFP vector (Maxygen, Palo Alto, CA, USA) with *Xba*I and *Cla*I and filling in the *Xba*I cut ends with Klenow DNA polymerase. The resulting 746-bp fragment was cloned into the *Cla*I and Klenow-filled *Hin*dIII sites downstream of the CMV promoter in pCLNCX (Crameri et al, 1996; Naviaux et al, 1996). Amphotropic retrovirus was produced by co-transfecting 2 × $10^6$ late-passage 293 cells with 20 μg of vector, either pCLNCX or pCLNCGFP, and the pCL-Ampho packaging-vector as described by Naviaux et al (1996). Viral supernatant was harvested 24 and 48 h after transfection. Viral titres were determined on BALB/c 3T3 cells by geneticin-resistant colony formation. HCT116 cells were infected with viral supernatant three times over a 12-h period in the presence of polybrene (8 μg $ml^{-1}$). Infected cells were selected for 9 days with geneticin (400 μg $ml^{-1}$) and the resulting population was identified as HCT116-GFP; GFP was expressed in high levels in 90–95% of these cells. Cells were subjected to flow cytometric analysis on a Becton Dickinson FACScan using an argon ion laser tuned to 488 nm to identify GFP-positive cells. Fluorescence was observed with a 515/545 bandpass filter.

### Enrichment assays

HCT116 + ch3 and HCT116-GFP cells were mixed in a 50:50 ratio and analysed by flow cytometry to document that the population contained 50% GFP-expressing cells. Enrichment assays were performed by plating 200 000 cells into 100-mm tissue culture dishes. After 24 h, an $IC_{50}$ concentration of each drug was added to the dishes, and the cells were exposed for 1 h (cisplatin, carboplatin), for 24 h (etoposide, melphalan, paclitaxel, perfosfamide, 6-thioguanine) or continuously (doxorubicin, tamoxifen). Flow cytometric analysis was performed 5 days later. Each experiment was performed three separate times for each drug. The HCT116 and HCT116-GFP cells, as well as the HCT116 cells infected with an empty pCL vector, were tested by clonogenic assay, and no difference in sensitivity to cisplatin or paclitaxel was found (data not shown).

### Data analyses

Mean ± s.d. values are indicated for all data sets. Two-sided *t* tests were performed to compare the effect of hMLH1 loss on drug sensitivity. Factorial ANOVA was used to analyse the extent of enrichment during treatment; *P*-values were calculated using the Scheffé procedure.

### RESULTS

### Cytotoxicity assays

The DNA mismatch repair-deficient HCT116 + ch2 cell line is known to be resistant to 6-thioguanine (Griffin et al, 1994), MNNG (Kat et al, 1993) and cisplatin and carboplatin (Anthoney et al, 1996; Fink et al, 1996). Figure 1 shows the survival curves for the mismatch repair-deficient HCT116 + ch2 cell line as well as the

© Cancer Research Campaign 1998

repair-proficient HCT116 + ch3 cell line as a function of drug concentration for doxorubicin, etoposide, perfosfamide, tamoxifen and 6-thioguanine. In comparison with the HCT116 + ch3 cell line, the DNA mismatch repair-deficient cells were also resistant with the topoisomerase II inhibitors etoposide and doxorubicin. The $IC_{50}$ values for all the drugs tested are presented in Table 1. The hMLH1-deficient HCT116 + ch2 cells were 2.0-fold more resistant to etoposide than the mismatch repair-proficient HCT116 + ch3 cells ($IC_{50}$, 0.73 ± 0.27 vs 0.36 ± 0.17 μM s.d.; $n = 3$; $P < 0.05$ in a two-sided *t*-test). Likewise, the mismatch repair-deficient cells were 1.7-fold more resistant to doxorubicin ($IC_{50}$, 16.1 ± 2.0 vs 9.3 ± 1.1 nM s.d.; $n = 3$; $P < 0.05$ in a two-sided *t*-test). The cytotoxicity of melphalan, paclitaxel, perfosfamide and tamoxifen was not affected by the mismatch repair status of the cells.

### Enrichment assays

Parental DNA mismatch repair-deficient HCT116 cells were infected with a retrovirus encoding the *GFP* gene driven by a CMV promoter, and a population that stably expressed GFP was selected. A population containing 50% DNA mismatch repair-deficient GFP-expressing cells and 50% repair-proficient HCT116 + ch3 cells was prepared by mixing and subjected to drug exposure. Five days later, the population was analysed by flow cytometry to document the proportion of GFP-expressing mismatch repair-deficient cells. Table 2 demonstrates the effect of loss of DNA mismatch repair on enrichment after a 1-h drug exposure (cisplatin, carboplatin), a 24-h drug exposure (etoposide, melphalan, paclitaxel, perfosfamide, 6-thioguanine), or a continuous drug exposure (doxorubicin, tamoxifen). Five days after a single exposure to an $IC_{50}$ concentration of etoposide, the treated population contained 44% more GFP-expressing mismatch repair-deficient cells than the untreated population. Likewise, the tumour cell population contained 36% more mismatch repair-deficient cells after a single exposure to doxorubicin. Thus, treatment with doxorubicin or etoposide, to which the DNA mismatch repair-deficient cells were twofold resistant, resulted in rapid enrichment of the population for the resistant cells.

### DISCUSSION

Loss of DNA mismatch repair can impact on the responsiveness of a tumour to chemotherapy in several different ways. First, loss of mismatch repair produces high-level resistance to the antimetabolite 6-thioguanine (Griffin et al, 1994), moderate levels of resistance to the methylating agent MNNG (Kat et al, 1993) and low-level resistance to cisplatin and carboplatin (Anthoney et al, 1996; Fink et al, 1996). It has recently been reported that loss of mismatch repair also causes resistance to temozolomide, a methylating agent that forms adducts similar to those of MNNG (Liu et al, 1996). Second, the genomic instability that accompanies loss of DNA mismatch repair can increase the rate of mutation in the coding or regulatory sequences of other genes whose products may play central roles in determining tumour cell sensitivity to drugs.

It has been suggested that the DNA mismatch repair system serves as a detector for the presence of DNA damage (Kat et al, 1993; Hawn et al, 1995). Resistance to 6-thioguanine, MNNG, temozolomide, cisplatin and carboplatin is thought to result from failure of the cell to recognize the DNA adducts formed by these drugs and to activate signalling pathways that trigger apoptosis. Indeed, pure hMSH2 has been reported to bind to platinated DNA in mobility shift assays (Mello et al, 1996), and human MutSα (Duckett et al, 1996), a heterodimer of hMSH2 and hMSH6 (Palombo et al, 1994; Acharya et al, 1996), has been shown to bind to DNA containing adducts produced by MNNG, 6-thioguanine and cisplatin. The molecular basis for the concept that loss of DNA mismatch repair causes resistance rather than hypersensitivity because the mismatch repair proteins serve as a detector of damage has been further substantiated by the recent observation that loss of mismatch repair results in failure to activate several cisplatin damage-responsive signal transduction pathways (Nehmé et al, 1997). In contrast, loss of mismatch repair does not result in resistance to oxaliplatin, and although little is known about the oxaliplatin adduct (Woynarowski et al, 1997), we have recently demonstrated that DNA adducts formed by oxaliplatin are not recognized by the mismatch repair system (Fink et al, 1996).

The results of both the cytotoxicity assays and enrichment assays reported here provide additional confirmation that loss of mismatch repair results in resistance to 6-thioguanine, cisplatin and carboplatin. As loss of DNA mismatch repair is not accompanied by resistance to the classical alkylating agents melphalan and perfosfamide, it is likely that the adducts produced by these agents are not recognized by the mismatch repair detector. Similarly, it has been reported that the adducts formed by the chloroethylating agent 1,3-bis(2-chloroethyl)-nitrosourea are not recognized by the mismatch repair complex (Liu et al, 1996). The lack of differential cytotoxicity of paclitaxel and tamoxifen is consistent with the fact that neither agent is known to interact with DNA at achievable clinical concentrations.

The results presented here extend the panel of drugs for which loss of mismatch repair causes resistance to the topoisomerase II inhibitors etoposide and doxorubicin. However, how loss of DNA mismatch repair produces low-level resistance to these agents is less clear than for those agents that react directly with DNA to produce adducts that distort its structure in a manner similar to that of true DNA mismatches. It may be that the mismatch repair proteins serve as a detector of the 'cleavable complex' (Chen et al, 1994) produced by the binding of etoposide or doxorubicin to topoisomerase II, or that the mismatch repair proteins normally act to stabilize the drug-induced 'cleavable complex' on the DNA, and thus serve to augment the DNA damage. Additional studies will be required to document interaction between the DNA mismatch repair proteins and topoisomerase II.

Several lines of evidence suggest that although loss of mismatch repair results in only relatively small degrees of resistance, this resistance is nevertheless of biological and clinical significance. First, even a twofold difference in sensitivity to cisplatin, carboplatin, doxorubicin and etoposide detected by clonogenic assays was sufficient to result in a clear enrichment for mismatch repair-deficient cells after only a single exposure of a proliferating tumour cell population to these drugs. Second, loss of DNA mismatch repair has been reported in tumour cell lines selected for resistance to cisplatin (Aebi et al, 1996) or doxorubicin (Drummond et al, 1996). Third, this laboratory has previously documented that only small degrees of resistance (< twofold) are required to account for clinical failure of cisplatin treatment (Andrews et al, 1990). $MSH2^{+/+}$ embryonic stem cells (de Wind et al, 1995) grown as xenografts were responsive to treatment with a single $LD_{10}$ dose of cisplatin, whereas isogenic $MSH2^{-/-}$ tumours were not, suggesting that the degree of cisplatin resistance conferred by loss of DNA mismatch repair is sufficient to produce a large difference in clinical responsiveness in vivo (Fink et al,

© Cancer Research Campaign 1998

1997). Because of the fact that embryonic stem cells require a drug-sensitive fibroblast feeder layer for prolonged propagation, studies of the extent to which low-level resistance can mediate enrichment for mismatch repair-deficient cells during treatment in vitro could not be addressed in this isogenic system, and we were limited to using the less truly isogenic HCT116 and HCT116 + ch3 pair of cells. Nevertheless, our results argue cogently that treatment with any of these five agents (carboplatin, cisplatin, doxorubicin, etoposide, 6-thioguanine) does select for repair-deficient cells, thus enriching the population. Additional studies are required to further document the kinetics of this process and the extent of enrichment that occurs in vivo with repeated cycles of drug exposure. However, it is likely that clinical resistance will become manifest at relatively low levels of enrichment. In mixing experiments performed with L1210 leukaemia cells sensitive and resistant to cyclophosphamide, Skipper et al (1978) demonstrated that the presence of only 1% resistant cells was sufficient to cause clinical failure of treatment.

The issue of when loss of mismatch repair occurs during oncogenesis remains controversial, even for hereditary non-polyposis colon cancer, which represents the best defined clinical situation (Tomlinson et al, 1996). However, once such cells are present in the tumour, their genomic instability may result in the accumulation of additional mutations that contribute to the phenomenon of tumour progression. Enrichment of these cells as a result of chemotherapy would be expected to accelerate this process. Indeed, Ben-Yehuda et al (1996) recently reported that microsatellite instability, a hallmark of the genomic instability due to the loss of mismatch repair (Loeb et al, 1994), was present in up to 94% of the patients with therapy-related leukaemia or myelodysplastic syndromes, consistent with drug-induced enrichment for genetically unstable cells.

The perfect concordance between the ability of a drug to enrich for GFP-expressing mismatch repair-deficient cells and loss of sensitivity to the same drug as assessed by clonogenic assay suggests that the former can be used as a way of rapidly screening for DNA mismatch repair-mediated changes in sensitivity to additional agents. The assay can be readily automated for high throughput screening, and the same principle can be used to examine the impact of the loss of other genes when isogenic pairs of cell lines are available.

### ACKNOWLEDGEMENTS

The authors would like to thank Drs C Richard Boland, Minoru Koi and Thomas A. Kunkel for generously making cell lines available. This work was supported in part by Fellowship Awards from the EMDO Stiftung, Zurich, and the Holderbank Stiftung to DF and from the Ernst Schering Research Foundation, Berlin, and the EMDO Stiftung, Zurich, to SN. This work was conducted in part by the Clayton Foundation for Research. SBH is a Clayton Foundation Investigator.

### REFERENCES

Acharya S, Wilson T, Gradia S, Kane MF, Guerrette S, Marsischky GT, Kolodner R and Fishel R (1996) hMSH2 forms specific mispair-binding complexes with hMSH3 and hMSH6. *Proc Natl Acad Sci USA* **93**: 13629–13634

Aebi S, Kurdi-Haidar B, Gordon R, Cenni B, Zheng H, Fink D, Christen RD, Boland CR, Koi M, Fishel R and Howell SB (1996) Loss of DNA mismatch repair in acquired resistance to cisplatin. *Cancer Res* **56**: 3087–3090

Aebi S, Fink D, Gordon R, Kim HK, Zheng H, Fink JL and Howell SB (1997) Resistance to cytotoxic drugs in DNA mismatch repair-deficient cells. *Clin Cancer Res*. **3**: 1763–1767

Andrews PA, Jones JA, Varki NM and Howell SB (1990) Rapid emergence of acquired *cis*-diamminedichloroplatinum(II) resistance in an *in vivo* model of human ovarian carcinoma. *Cancer Commun* **2**: 93–100

Anthoney DA, McIlwrath AJ, Gallagher WM, Edlin ARM and Brown R (1996) Microsatellite instability, apoptosis, and loss of p53 function in drug-resistant tumor cells. *Cancer Res* **56**: 1374–1381

Ben-Yehuda D, Krichevsky S, Caspi O, Rund D, Polliack A, Abeliovich D, Zelig O, Yahalom V, Paltiel O, Or R, Peretz T, Ben-Neriah S, Yehuda O and Rachmilewitz EA (1996) Microsatellite instability and p53 mutations in therapy-related leukemia suggest mutator phenotype. *Blood* **88**: 4296–4303

Bhattacharyya NP, Skandalis A, Ganesh A, Groden J and Meuth M (1994) Mutator phenotypes in human colorectal carcinoma cell lines. *Proc Natl Acad Sci USA* **91**: 6319–6323

Boyer JC, Umar A, Risinger JI, Lipford JR, Kane M, Yin S, Barrett JC, Kolodner RD and Kunkel TA (1995) Microsatellite instability, mismatch repair deficiency, and genetic defects in human cancer cell lines. *Cancer Res* **55**: 6063–6070

Chen AY and Liu LF (1994) DNA topoisomerases: essential enzymes and lethal targets. *Ann Rev Pharmacol Toxicol* **36**: 191–218

Crameri A, Whitehorn EA, Tate E and Stemmer WPC (1996) Improved green fluorescent protein by molecular evolution using DNA shuffling. *Nature Biotechnol* **14**: 315–319

de Wind N, Dekker M, Berns A, Radman M and te Riele H (1995) Inactivation of the mouse *Msh2* gene results in mismatch repair deficiency, methylation tolerance, hyperrecombination, and predisposition to cancer. *Cell* **82**: 321–330

Drummond JT, Anthoney A, Brown R and Modrich P (1996) Cisplatin and adriamycin resistance are associated with MutLα and mismatch repair deficiency in an ovarian tumor cell line. *J Biol Chem* **56**: 19645–19648

Duckett DR, Drummond JT, Murchie AIH, Reardon JT, Sancar, A., Lilley DMJ and Modrich P (1996) Human MutSα recognizes damaged DNA base pairs containing $O^6$-methylguanine, $O^4$-methylthymine, or the cisplatin-d(GpG) adduct. *Proc Natl Acad Sci USA* **93**: 6443–6447

Fink D, Nebel S, Aebi S, Zheng H, Cenni B, Nehmé A, Christen RD and Howell SB (1996) The role of DNA mismatch repair in platinum drug resistance. *Cancer Res* **56**: 4881–4886

Fink D, Zheng H, Nebel S, Norris PS, Aebi S, Lin TP, Nehmé A, Christen RD, Haas M, MacLeod CL and Howell SB (1997) *In vitro* and *in vivo* resistance to cisplatin in cells that have lost DNA mismatch repair. *Cancer Res* **57**: 1841–1845

Fishel R and Kolodner RD (1995) Identification of mismatch repair genes and their role in the development of cancer. *Curr Opin Genet Dev* **5**: 382–395

Griffin S, Branch P, Xu YZ and Karran P (1994) DNA mismatch binding and incision at modified guanine bases by extracts of mammalian cells: implications for tolerance to DNA methylation damage. *Biochemistry* **33**: 4787–4793

Hawn MT, Umar A, Carethers JM, Marra G, Kunkel TA, Boland CR and Koi M (1995) Evidence for a connection between the mismatch repair system and the $G_2$ cell cycle checkpoint. *Cancer Res* **55**: 3721–3725

Huang J, Papadopoulos N, McKinley AJ, Farrington SM, Curtis LJ, Wyllie AH, Zheng S, Willson JKV, Markowitz SD, Morin P, Kinzler KW, Vogelstein B and Dunlop MG (1996) *APC* mutations in colorectal tumors with mismatch repair deficiency. *Proc Natl Acad Sci USA* **93**: 9049–9054

Kat A, Thilly WG, Fang WH, Longley MJ, Li GM and Modrich P (1993) An alkylation-tolerant, mutator human cell line is deficient in strand-specific mismatch repair. *Proc Natl Acad Sci USA* **90**: 6424–6428

Koi M, Umar A, Chauhan DP, Cherian SP, Carethers JM, Kunkel TA and Boland CR (1994) Human chromosome 3 corrects mismatch repair deficiency and microsatellite instability and reduces *N*-methyl-*N'*-nitro-*N*-nitrosoguanidine tolerance in colon tumor cells with homozygous *hMLH1* mutation. *Cancer Res* **54**: 4308–4312

Kolodner R (1996) Biochemistry and genetics of eukaryotic mismatch repair. *Genes Dev* **10**: 1433–1442

Liu L, Markowitz S and Gerson SL (1996) Mismatch repair mutations override alkyltransferase in conferring resistance to temozolomide but not to 1,3-bis(2-chloroethyl)nitrosourea. *Cancer Res* **56**: 5375–5379

Loeb LA (1994) Microsatellite instability: marker of a mutator phenotype in cancer. *Cancer Res* **54**: 5059–5063

Markowitz S, Wang J, Myeroff L, Parsons RE, Sun L, Lutterbaugh J, Fan RS, Zborowska E, Kinzler KW, Vogelstein B, Brattain M and Willson JKV (1995) Inactivation of the type II TGF-β receptor in colon cancer cells with microsatellite instability. *Science* **268**: 1336–1338

© Cancer Research Campaign 1998

Mello JA, Acharya S, Fishel R and Essigmann JM (1996) The mismatch-repair protein hMSH2 binds selectively to DNA adducts of the anticancer drug cisplatin. *Chem Biol* **3**: 579–589

Morin J and Hastings J (1972) Energy transfer in a bioluminescent system. *J Cell Physiol* **77**: 313–318

Naviaux RK, Costanzi E, Haas M and Verma IM (1996) The pCL vector system: rapid production of helper-free, high-titer, recombinant retroviruses. *J Virol* **70**: 5701–5705

Nehmé A, Baskaran R, Aebi S, Fink D, Nebel S, Cenni B, Wang JYJ, Howell SB and Christen RD (1997) Differential induction of c-Jun $NH_2$-terminal kinase and c-Abl kinase in DNA mismatch repair-proficient and -deficient cells exposed to cisplatin. *Cancer Res* **57**: 3253–3257

Palombo F, Gallinari P, Iaccarino I, Lettieri T, Hughes M, D'Arrigo A, Truong O, Hsuan JJ and Jiricny J (1995) GTBP, a 160-kilodalton protein essential for mismatch-binding activity in human cells. *Science* **268**: 1912–1914

Skipper HE, Schabel FM and Lloyd HH (1978) Experimental therapeutics and kinetics: selection and overgrowth of specifically and permanently drug-resistant tumor cells. *Semin Hematol* **15**: 207–219

Tomlinson IPM, Novelli MR and Bodmer WF (1996) The mutation rate and cancer. *Proc Natl Acad Sci USA* **93**: 14800–14803

Woynarowski JM, Chapman WG, Napier C and Raymond E (1997) Oxaliplatin (OxPt) effects on naked and intracellular DNA (abstract). *Proc Am Assoc Cancer Res* **38**: 311

© Cancer Research Campaign 1998